# Inflammatory protein signatures as predictors of insulin resistance and cognitive function: a one-year longitudinal study in the *m*PRIME cohort

**DOI:** 10.1016/j.bbih.2026.101366

**Published:** 2026-09-22

**Authors:** Carmen Schiweck, Judith Gruber, Janita Bralten, Giuseppe Fanelli, Indira Paz-Graniel, Veerle van Gils, Carlos Gomez-Martinez, Jordi Salas-Salvadó, Jeffrey Glennon, Geert Poelmans, Barbara Franke, Andreas Reif, Silke Matura

**Affiliations:** a Department of Psychiatry, Psychotherapy and Psychosomatics, Goethe University Frankfurt, Germany; b Donders Institute for Brain, Cognition and Behaviour, Radboud University, Nijmegen, the Netherlands; c Department of Human Genetics, Radboud University Medical Center, Nijmegen, the Netherlands; d Department of Biomedical and Neuromotor Sciences, University of Bologna, Bologna, Italy; e Department of Medical Neuroscience, Radboud University Medical Center, Nijmegen, the Netherlands; f Universitat Rovira i Virgili, Departament de Bioquímica i Biotecnologia, Alimentació, Nutrició, Desenvolupament i Salut Mental ANUT-DSM, Reus, Spain; g Alimentació, Nutrició, Desenvolupament i Salut Mental, Institut d’Investigació Sanitària Pere Virgili (IISPV), Reus, Spain; h Centro de Investigación Biomédica en Red Fisiopatología de La Obesidad y Nutrición (CIBEROBN), Instituto de Salud Carlos III, Madrid, Spain; i Alzheimer Center Limburg, Mental Health and Neuroscience Research Institute, Maastricht University, Maastricht, the Netherlands; j Conway Institute of Biomolecular and Biomedical Research, School of Medicine, Belfield, Dublin 4, Ireland; k Division of Psychiatry, Haukeland University Hospital, Bergen, Norway; l Department of Biomedicine, University of Bergen, Bergen, Norway; m Fraunhofer Institute for Translational Medicine and Pharmacology ITMP, Frankfurt am Main, Germany

## Abstract

**Background:**

Large-scale studies have demonstrated a temporal relationship between insulin resistance (IR) and peripheral inflammation, with emerging evidence also suggesting a link to cognitive decline. However, most of the existing research has focused on individual inflammatory proteins, while the role of inflammatory protein signatures associated with IR and cognition remains unexplored.

**Aim:**

In this study, we investigated a) whether specific inflammatory protein signatures are associated with IR; b) whether baseline inflammatory protein signatures predict changes in Homeostatic Model Assessment for IR (HOMA-IR) after one-year follow-up, and c) whether baseline IR and/or specific inflammatory signatures predicted cognitive performance at follow-up.

**Methods:**

In 110 healthy adults from the *m*PRIME study, 45 inflammatory markers (measured by OLINK Proximity Extension Assay), HOMA-IR (as an index of IR), and cognitive function (as measured with an extensive neuropsychological test battery) were assessed at baseline and one-year follow-up. Inflammatory protein signatures were defined using hierarchical cluster analysis. Regression and cross-lagged analyses examined relationships between inflammation, HOMA-IR and cognitive changes over time.

**Results:**

Next to the cumulative score, two inflammatory protein signatures were identified, related to 1) cytokine signalling, 2) immune cell migration. At baseline, the cytokine signalling signature correlated with HOMA-IR; together with BMI, CCL7 and CCL4 were consistently selected in >99% of models, with the penalised regression signature explaining 38,2% of HOMA-IR variance (mean out of sample R^2^). Cross-lagged analyses showed that baseline HOMA-IR predicted immune cell migration and IL-6 levels at follow-up, while CRP predicted future HOMA-IR. Cognitive performance (global cognitive performance, working memory z scores but not executive functioning) were nominally associated with the cytokine signalling signature and baseline HOMA-IR but did not survive FDR correction. No consistent association was found for inflammation, HOMA-IR and the one-year change in cognitive performance.

**Conclusion:**

Our findings contribute to a better understanding of the specific inflammatory proteins involved in the temporal association between IR and inflammation: while CRP predicts HOMA-IR, the more specific signature linked to cytokine-signalling did not; instead, a high HOMA-IR predicted higher inflammation related to immune cell migration. We discovered proteins including CCL4, CCL7 and IL-10 associated with HOMA-IR, which should be explored further.

## 1 Introduction

The societal shift to unhealthy, high-fat, high-sugar diets contributes to insulin resistance (IR). Obesity rates rose drastically in the past 20 years, with around 1 billion adult males and 1.11 billion adult females suffering from obesity (i.e., a 155% increase for males and 104% for females compared to 1991) (Ng et al., 2025). The prevalence of IR is estimated at around 20-40% in the general population (Qu et al., 2011; Friedrich et al., 2012), with higher rates in obesity (Blüher, 2010).

IR is the reduced capability of cells to respond to insulin, particularly in skeletal muscle, adipose tissue, and the liver (Lee et al., 2021), leading to impaired glucose uptake and metabolism, and eventually to type 2 diabetes mellitus (T2DM). Several factors contribute to the onset of IR. Best known are lifestyle and dietary choices (Reis et al., 2011), stress, medications such as corticosteroids (which can disrupt glucose homeostasis directly or through weight gain) (Fève and Scheen, 2022), and genetic predisposition (Watanabe et al., 2019; Lind, 2019). In the past decades, inflammation has also been identified as a risk and/or maintenance factor for IR, and it is in itself linked to many of the above-mentioned risk factors (Garcia et al., 2010).

When adipose tissue expands (e.g., due to an unhealthy diet), increased local infiltration of macrophages and other immune cells into tissues can lead to an inflammatory milieu and IR (Khan et al., 2015). The continuous secretion of inflammatory proteins and proliferation of macrophages sustains adipose tissue inflammation (Zamarron et al., 2016) and can exacerbate inflammatory states (Rohm et al., 2022; Wu and Ballantyne, 2020). Additionally, it has been shown that hyperinsulinemia alone is sufficient to trigger adipose tissue inflammation (Pedersen et al., 2015), and some evidence suggests that tissue-specific IR may precede immune cell accumulation in cases of diet-induced IR (Shimobayashi et al., 2018). To identify proteins related to this course, previous studies have mostly focussed on linking single inflammatory proteins (i.e., cytokines, chemokines) to T2DM, showing an association between some, but not all cytokines. For instance, while higher levels of IL-6 were strongly correlated with T2DM, the results for TNF-alpha, IL1β and IL-18 were less robust (Liu et al., 2016). Next to IL-6, the most robust finding to date is the positive association between C-reactive protein (CRP) and IR, observed both cross-sectionally and longitudinally (Liu et al., 2016; Park et al., 2009). Strikingly, elevated high-sensitivity CRP (hsCRP) has been associated with an increased risk of future IR, even in individuals free from diabetes (Yan et al., 2019), with large-scale studies in over 6000 children and 6000 adults supporting this association (Palmer et al., 2024; Lim and Lee, 2024; Nagrani et al., 2022).

Despite the ongoing research into the role of inflammation in IR and vice versa, it is still not entirely clear which of them precedes the other; partly due to the samples already comprising people with T2DM, partly because the focus often lies on single inflammatory proteins that are easily measurable in the central laboratory (i.e., such as CRP or IL-6). Yet, identifying both the temporal sequence and specific proteins could help in identifying suitable treatment targets. Indeed, several animal- and some human studies have explored preventing or delaying IR by reducing inflammation: Experiments in mice have shown that inhibiting inflammatory signalling by knocking out key pathways (e.g., NLRP3) can indeed prevent the development of IR and improve insulin sensitivity (Chiazza et al., 2015) and first studies have shown promise for some, but not all anti-inflammatory agents (Rohm et al., 2022). To improve treatment effectiveness, a crucial step is to identify the temporal association of inflammation not only for single proteins, but to identify protein signatures that could yield more insight and may in the future, be used as biomarkers (for disease states and/or as treatment predictors). However, while several proteomic studies exist for insulin resistant samples, proteomic analyses investigating the moderating effect between IR and several inflammatory protein biomarkers and their link to cognition are, though rising, still limited (Zanetti et al., 2023). The technologically more refined proteomic platforms that are nowadays available, facilitate the investigation of protein co-expression in more detail and could help speed up the process (Nowak et al., 2015).

The relationship between IR and inflammation is not only relevant for the periphery, but also for the brain: it is well known that people with T2DM are at higher risk for dementia and cognitive decline, and more recently awareness of the importance of treating this comorbidity has risen (Srikanth et al., 2020). Mechanistically, circulating cytokines, hyperglycaemia and oxidative stress can together reduce tight-junction integrity and increase blood–brain barrier (BBB) permeability (e.g. (Erickson and Banks, 2018)). In turn, higher permeability can facilitate inflammatory signalling between the periphery and CNS. Evidence from human gadolinium-enhanced MRI studies have shown increased BBB permeability in type 2 diabetes, including increased contrast accumulation in the basal ganglia (Qiao et al., 2026; Starr et al., 2003). Such BBB dysfunction may in turn promote glial activation and central insulin resistance, consistent with MRI evidence linking hypothalamic gliosis to higher fasting insulin and HOMA-IR in humans (Schur et al., 2015). Furthermore, Qiao et al. (2026) recently used dynamic contrast-enhanced MRI in 75 individuals with T2DM. Within this group, when comparing those with and without cognitive impairment (based on the Montreal Cognitive Assessment) they found increased BBB permeability in individuals with T2DM and cognitive impairment (Qiao et al., 2026). Importantly, the authors also found functional consequences of these alterations, with greater permeability in the basal ganglia and hippocampus being associated with poorer cognitive performance, even after adjustment for relevant covariates (Qiao et al., 2026). Taken together, these findings provide a biological rationale for our hypothesis that inflammation may moderate the association between IR and cognition, as inflammatory processes may exacerbate BBB dysfunction and thereby increase vulnerability to the adverse cerebral effects of IR.

Interestingly, on a behavioural level, *both* inflammation *and* IR have been linked to worse cognitive performance: Previous cross-sectional studies have already established an association between HOMA-IR and cognitive performance (Sherzai et al., 2018). Others have also shown an association of cognitive performance with HOMA-IR in prospective, longitudinal studies (e.g. (Ekblad et al., 2017; Kong et al., 2018; Ennis et al., 2020)). Similarly, it has been shown that higher levels of inflammation, as measured with cytokines including IL-6, TNF-alpha and hs-CRP are linked to worse cognitive performance and mild cognitive impairment (Du et al., 2023; Kipinoinen et al., 2022a; Beydoun et al., 2018). To illustrate, it was found that inflammatory markers such as IL-6 predicted cognitive performance and decline over a 10-year follow-up period (Kipinoinen et al., 2022b), particularly for executive functioning and processing speed (Sluiman et al., 2022). Given this association, it is possible that the relationship between IR and cognitive impairment is moderated by inflammation, but the combined effect of inflammation and IR on cognitive capacity has, to the best of our knowledge less often been tested formally using proteomic data. Therefore, in this secondary analysis of the *m*PRIME cohort we set out to a) explore individual protein signatures and potential mechanisms associated with IR; b) explore their temporal dynamics during a one-year follow-up period and, c) assess the moderating effect of inflammatory signatures on IR (as indexed by the Homeostatic Model Assessment for Insulin Resistance (HOMA-IR)) for cognitive performance and d) cognitive decline after one year.

## 2 Methods

### 2.1 Ethical approval & informed consent

The study was performed at the Department of Psychiatry of the University Hospital Frankfurt, Germany, as part of the *m*PRIME project, https://prime-study.eu/) and approved by the local research ethics committee (ethics approval number 20-767). The study was registered in the German Clinical Trials Register (DRKS00022774). Written informed consent was obtained from all participants prior to any study-related procedures.

### 2.2 Participants

Between December 2020 and March 2023, 124 individuals were enrolled. Six dropped out due to the substantial time commitment required for participation; 1 individual was excluded from the analysis due to a Mini-Mental State Examination (MMSE) score <25, 1 individual was excluded because of an existing untreated type 2 diabetes without meeting the criteria for IR, leaving 116 participants for inclusion. Participants were ≥18 years and an effort was made to recruit people with a body mass index (BMI) ≥24 kg/m^2^, as defined in the inclusion criteria. Exclusion criteria included: diagnosis of diabetes mellitus other than type 2 (e.g., type 1 or gestational diabetes); use of oral antidiabetic medications or insulin, glucocorticoids, or weight-loss drugs; special dietary regimens; neurological diseases; a diagnosis of bipolar I disorder, schizophrenia, organic mental disorders, or substance abuse; current pregnancy or breastfeeding. Individuals were also excluded from participation if they missed the first appointment, had insufficient knowledge of German or English, or performed poorly on cognitive tests (MMSE<25 and/ora score of <75 on the Multiple-Choice Vocabulary Intelligence Test at screening). Participants received one-time, personalised feedback on their diet, exercise, cognitive performance, and blood glucose levels after the ambulatory assessment as per protocol. Additionally, they were offered an incentive payment and invited to participate in a biannual voucher raffle.

### 2.3 Procedure

The study protocol has been described in detail elsewhere (Gruber et al., 2024). In brief, at baseline, all participants were screened for psychiatric diagnoses using the MINI Diagnostic Interview (Sheehan et al., 1998). After inclusion, all study individuals completed questionnaires on sociodemographic characteristics, lifestyle, a measurement of BMI, waist circumference, and a neuropsychological assessment. Relevant measures included the Beck's Depression Inventory (BDI-II), the Rey-Osterrieth Complex Figure test, phonemic and semantic verbal fluency tests - specifically, letter “S” and the category “animals”, Trail Making Test A (TMT-A) and B (TMT-B), Digit Span (Forward and Backward), Block Span (Forward and Backward), and Letter-Number Span (Gruber et al., 2024). Cognitive performance was evaluated for three domains (Gruber et al., 2024): global cognition (GC), working memory (WM), and executive function (EF). The WM domain score incorporated results from the Digit Span Forward and Backward, Block Span Forward and Backward, and Letter-Number Span tests. EF was evaluated through phonemic and semantic verbal fluency tests and TMT-B. All test scores were transformed to z-scores to aid in interpretation, with TMT-A and B z-scores inverted so that higher scores indicated better performance. A global cognitive z-score (GC) was created by averaging individual z-scores from each test, always using the mean and standard deviation at baseline. WM and EF z-scores were obtained by using their respective cognitive tests. After one year, participants were invited to repeat the same measures. An overview of the *m*PRIME study design is shown in Fig. 1.

**Fig. 1:**
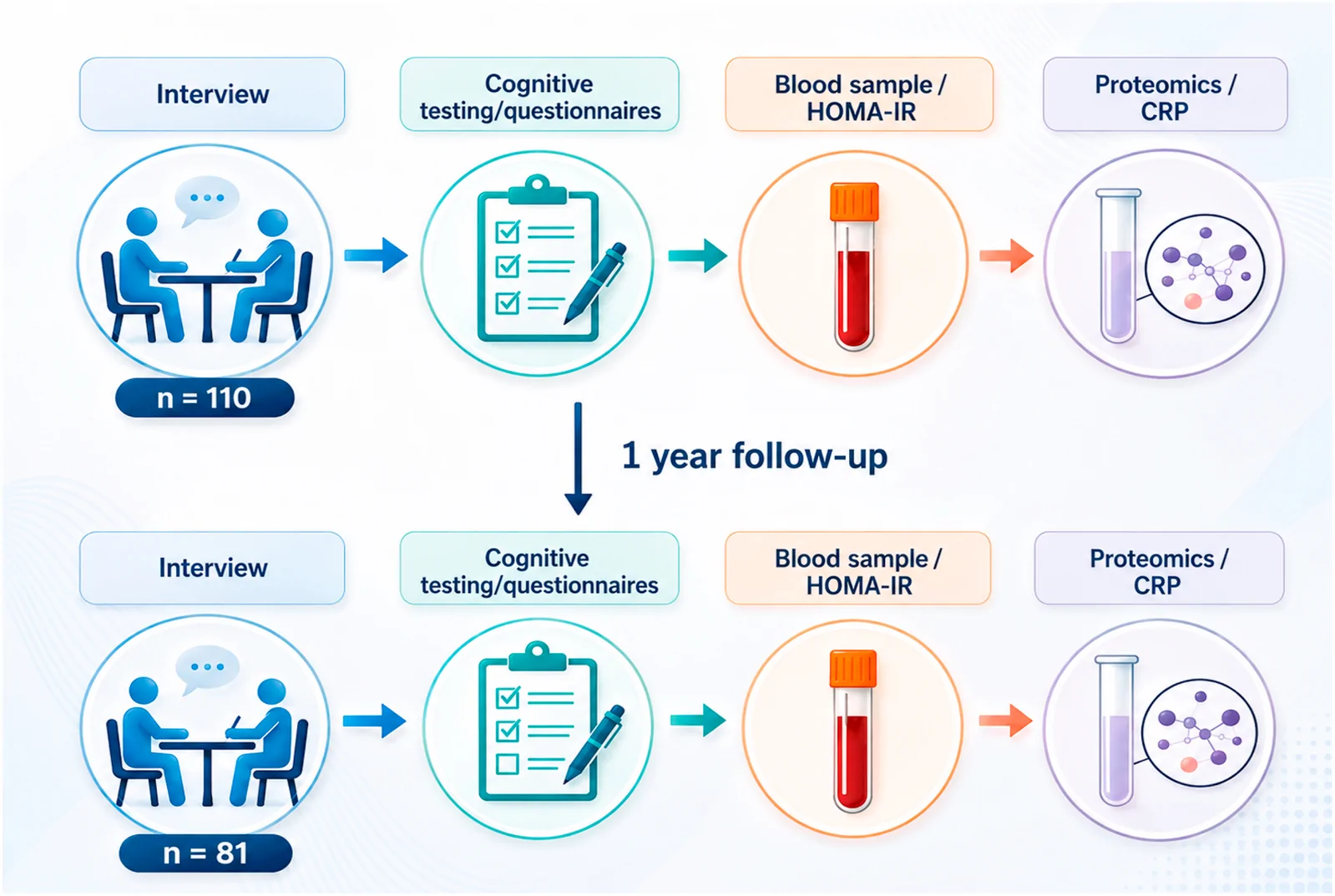
Study Design of the *m*PRIME study with relevant elements for the current report.

### 2.4 Blood biomarkers

At baseline and one-year follow-up, blood samples were taken after an overnight fast (>8h), and collected in K3EDTA Monovettes (Sarstedt, Germany) for plasma storage and future Olink analyses, and in GLP plasma tubes (Sarstedt Germany) for glucose, insulin and Hba1c measurement.

### 2.5 Proximity Extension Assay

Proteomic analysis was performed using the Olink Target 48 panel (Olink Proteomics, Uppsala, Sweden) (Olink Proteomics and Uppsala, 2023), which quantifies 45 proteins related to inflammatory processes via Proximity Extension Assay (PEA) technology. In contrast to standard OLINK panels, this panel provides absolute concentrations of proteins (not NPX values) which were further used for analysis. Of note for analysis, proteins were standardized (mean = 0, SD = 1) and transformed where applicable (see statistic section below). Furthermore, longitudinal samples from the same participant were analysed on the same plate. Five plates from a single LOT were reserved for the study, and Olink Target 48 data were normalized using the plate-specific calibrators within the Olink software, thereby minimizing plate-, lot-, and batch-related variability.

### 2.6 Homeostatic model assessment of insulin resistance (HOMA-IR) determination

The HOMA-IR index was calculated as a proxy for IR, using fasting glucose and insulin levels according to the standard hospital procedures and with the formula (Matthews et al., 1985): $\text{HOMA}‐\text{IR}=\frac{\text{fasting}\;\text{blood}\;\text{glucose}\;\left(\text{mg}/\text{dl}\right)\;x\;\text{fasting}\;\text{blood}\;\text{insulin}\;\left(\text{mcU}/\text{ml}\right)}{405}\;\text{.}$ In addition, Hba1c was measured in the central laboratory of the university clinic.

### 2.7 Statistical analysis

Pre-processing consisted of imputation of missing values, where appropriate, and checking variables for normality of distribution and homogeneity of variance. Due to the nature of the protein data and HOMA-IR, which had a slight to considerable deviance from a normal distribution, data was transformed using the best out of eight different power transformations (i.e., arcsinh(x), Box-Cox, Center + scale, Double Reversed Log_b(x+a), Exp(x), Log_b(x+a), sqrt(x + a) or Yeo-Johnson transformation), available in the *bestNormalize* package in R (Peterson and Peterson, 2020), which was able to restore approximate normality and improved homogeneity of variance. For logarithmic or square-root transformations of variables containing non-positive values, a constant was added such that the minimum shifted value equalled 1, thereby avoiding extreme transformed values resulting from observations close to zero.

#### 2.7.1 Protein signatures

Of the 45 proteins analysed with OLINK, nine were missing more than 40% of values, and were excluded from analyses (IL-1B, Il-13, CSF-2, IL-4, TLSP, IL-33, IL-2, IL-17A, IL-17F), leaving 36 proteins for further analysis. For two additional proteins missing 1% of data each (EFG and CXCL11), the assay indicated that the missing values were above the upper limit of quantification. These missing values were replaced by the upper level of quantification. No other imputation took place. Inflammatory protein signatures were based on hierarchical clustering. Protein concentrations were standardised (mean = 0, SD = 1), and Euclidean distances between protein profiles were calculated. Hierarchical clustering was performed using Ward's minimum variance method (Ward.D2). Candidate solutions with two to seven clusters were evaluated using the gap statistic, mean silhouette width, and bootstrap stability. To assess the robustness of the cluster structure, participants were resampled with replacement 1000 times; protein concentrations were re-standardised and the hierarchical clustering procedure repeated within each bootstrap sample. Stability of each original protein cluster was quantified by its maximum Jaccard similarity with the corresponding bootstrap clusters. Standardised protein profiles and the resulting hierarchical clustering were visualised using the ComplexHeatmap package (Gu, 2022).

Next, protein connections and functions were explored in the STRING-db web-based application (v12.5, https://string-db.org/), applying standard settings (i.e., using a full STRING network, medium confidence score for the interactions set to 0.400, interactors using query proteins only). The top three biological functions based on the false discovery rate (FDR) of the functional enrichment analysis for each cluster were selected for description of cluster functions.

#### 2.7.2 Cumulative inflammatory score calculation

A cumulative inflammatory score was calculated by addition of all standardized protein concentrations within one cluster (Schnorr et al., 2024). To do so, internal consistency of proteins within one cluster was assessed using Cronbach's alpha, with proteins scoring <0.7 excluded. Negatively correlated proteins were reverse scored before addition. In addition to the overall composite score containing all proteins, sub-scores for each of the three clusters were calculated. Given that Cronbach's alpha was above 0.7 for all proteins, no protein was dropped. Given that we were also interested in changes over time, the same calculation was used for the follow-up timepoint. Of note, given that the strongest evidence for inflammation and HOMA-IR exists for CRP (see introduction) and IL-6 is most frequently measured we also add separate analyses for those two proteins.

#### 2.7.3 Baseline analyses

Demographic characteristics were summarized for all participants. We assessed the association between inflammatory proteins and HOMA-IR levels (both treated as continuous variables). Cumulative scores and individual proteins were compared using separate MANCOVAs, with age, BMI and sex included as covariates). If Pillai's trace test was significant, follow-up ANCOVAs (type III, adjusting for age, BMI, and sex) were conducted, and FDR correction was applied (Benjamini Hochberg). Furthermore, we explored whether inflammatory proteins explained variance in HOMA-IR at baseline, beyond BMI, age, and sex. To do so, penalised regression was used to identify predictors of baseline HOMA-IR among age, sex, BMI, and the relevant proteins. All predictors were subject to penalisation. Model tuning was performed using repeated nested 10-fold cross-validation with 20 repetitions. Within each outer training fold, the elastic-net mixing parameter (α; 0.1–1.0 in increments of 0.1) and penalty parameter (λ) were selected by inner 10-fold cross-validation using the mean squared error. Predictive performance was evaluated in the held-out outer folds and summarized using cross-validated R^2^, root mean squared error (RMSE), and mean absolute error (MAE). Predictor stability was quantified as the proportion of outer-loop models in which each variable had a non-zero coefficient. Mean penalised coefficients were additionally calculated across the repeated outer-loop models. The analysis was considered exploratory given the modest sample size.

#### 2.7.4 Longitudinal prediction - cross lagged panel analysis

To assess whether baseline inflammation could predict IR (HOMA-IR) at follow-up, or vice versa, cumulative inflammatory scores and HOMA-IR were used in a cross lagged panel analysis, using the *lavaan* package in R (Rosseel, 2011). Additionally, an exploratory analysis was conducted, using all proteins in a significant cluster to assess whether a single protein also had predictive value. Path coefficients were visualised using the *semPlot* package (Epskamp, 2015).

#### 2.7.5 Cognitive performance analyses

Correlation analyses (simple and partial correlation with age and education as covariates) were conducted using Pearson's method to examine whether there was an association between baseline inflammation/HOMA-IR and cognitive performance. To assess the effect of single inflammation scores and HOMA-IR on cognitive decline, robust linear regression models (*lmrob* package (Maechler et al., 2021)) were used with the cognitive z-score at T2 as dependent variable and age, BMI, education (collapsed to 4 levels: any school degree, vocational education, undergraduate university degree, postgraduate university degree), as well as baseline cognitive z-score as covariates, the HOMA-IR and inflammation clusters as well as their interaction as predictors. Benjamini-Hochberg correction was applied.

## 3 Results

For a total of 110 participants, blood samples were available at baseline; 81 participants had samples at follow-up, and 79 participants had a sample at both timepoints. Characteristics of the study population are displayed in Table 1. HOMA-IR, insulin, hsCRP, and IL-6 did not change significantly between timepoints (paired Wilcoxon test all >0.1), while glucose (median baseline to 88 to 85 mg/dL, p = 0.031) and HbA1c (median baseline 5.49 to 1 year: 5.41, p = 0.004) levels decreased slightly. As expected, at baseline, HOMA-IR was very strongly correlated with fasting insulin (Spearman's ρ = 0.98, p < 0.001), fasting glucose (ρ = 0.55, p < 0.001) and HbA1c (ρ = 0.45, p < 0.001).

**Table 1:** **Baseline demographic data of the *m*PRIME sample available for this analysis.** Abbreviations: N = number, SD = standard deviation, HOMA-IR=Homeostatic Model Assessment for Insulin Resistance; CRP=C Reactive Protein, IL-6 = Interleukin 6, BDI-II= Becks's Depression inventory.

Demographics	N	Mean/SD, N (%)
Age [years]	110	50.18 (14.45)
Sex	110	
Female		65 (59%)
Male		45 (41%)
BMI	110	28.53 (6.06)
Smoking	110	
No		96 (87%)
Yes		8 (7.3%)
Occasionally		6 (5.5%)
HOMA-IR	109	2.82 (2.46)
HOMA-IR Group	109	
Insulin resistant		52 (48%)
Normal		57 (52%)
Insulin (μIU/ml)	109	12.03 (9.07)
Glucose (mg/dl)	109	90.38 (14.78)
HbA1c (%)	109	5.53 (0.44)
CRP [mg/dL]	109	0.24 (0.53)
IL-6 [pg/mL]	110	3.25 (3.59)
BDI-II	109	6.17 (6.64)
Education	110	
Basic/secondary school certificate (Hauptschule)		1 (0.9%)
Intermediate secondary school certificate (Realschule/Mittlere Reife)		11 (10%)
Grammar school diploma (Abitur)		12 (11%)
Completed vocational training		21 (19%)
University of Applied Sciences degree		8 (7.3%)
University degree (Bachelor)		12 (11%)
University degree (Master, Diploma)		39 (35%)
University doctoral degree (PhD)		6 (5.5%)
Contraception	35	
No		31 (89%)
Yes		4 (11%)

^*1*^ Mean (SD); n (%).

### 3.1 Inflammatory protein signatures

Bootstrap reproducibility was greatest for the two-cluster solution (mean Jaccard = 0.72; with cluster-specific mean Jaccard coefficients of 0.781 and 0.654, indicating moderate stability. Stability decreased for solutions with larger numbers of clusters (mean Jaccard coefficients: k = 3, 0.631; k = 4, 0.554; k = 5, 0.527; k = 6, 0.527; k = 7, 0.562). The resulting two clusters contained 25 and 11 proteins, respectively. The GAP statistic was slightly higher for the three-cluster solution (GAP = 0.308, SE = 0.020) than for the two-cluster solution (Gap = 0.284, SE = 0.020), whereas mean silhouette widths were low across all candidate solutions (0.11–0.15), indicating only modest separation between clusters. Given that the included proteins represent related inflammatory processes, complete separation between clusters was not assumed. The more parsimonious two-cluster solution was therefore retained based primarily on its greater bootstrap reproducibility. Due to the relatively small sample and the known sensitivity of hierarchical clustering to analytic specification, the resulting signatures were considered exploratory rather than definitive biological subprocesses.

The largest cluster 1 (see Fig. 2) contained most interleukins and chemokines: IFNG, MMP12, CXCL9, CXCL10, TGFA, CSF1, IL-10, IL-15, TNFSF10, LTA, CCL19, TNF, FLT3LG, IL-6, CSF3, CCL8, CCL11, CCL2, CXCL8 (IL-8), CCL7, IL-27, IL-17C, IL-18, CCL3 and CCL4. The protein cluster contained several cytokines with pro-inflammatory functions, as well as chemokines involved in attracting immune cells. Hereafter, we refer to this cluster as the “*cytokine signalling*” protein signature (Top 5 functions according to FDR: cytokine-mediated signalling pathway, inflammatory response, immune response, Leukocyte migration, response to other organism). Of note, the anti-inflammatory cytokine IL-10 was also included in this cluster. This synchronous upregulation of pro-and anti-inflammatory proteins is a commonly observed pattern in response to inflammation.

**Fig. 2:**
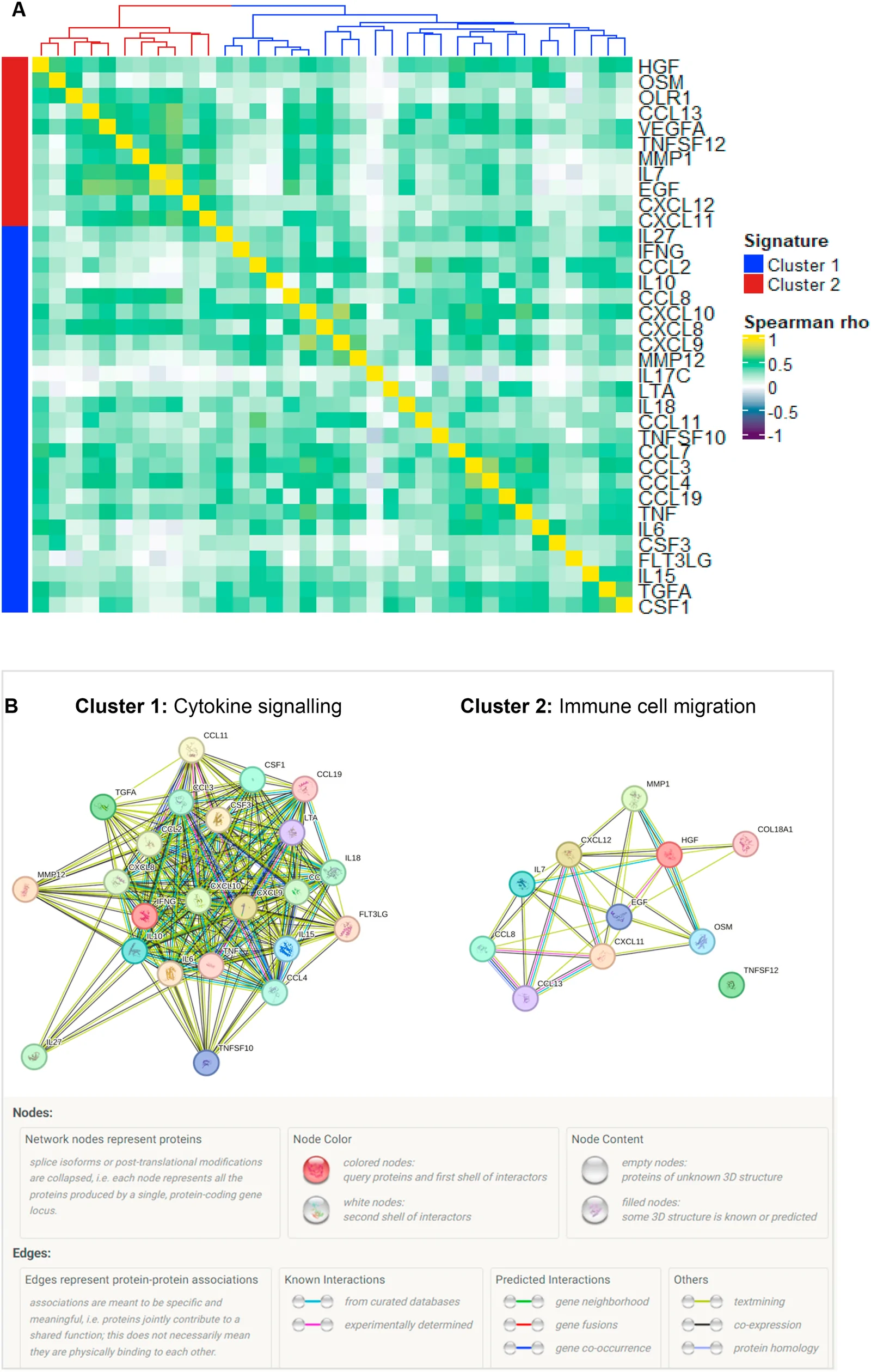
**Protein Clusters**. **A.** Heatmap of the two clusters used to calculate inflammatory sub scores for protein signatures. **B.** Graphical representation of protein networks.

Cluster 2, hereafter called “*immune cell migration*” protein signature, included HGF, IL-7, CXCL11, CXCL12, MMP1, TNFSF12-7, OSM, OLR-1, EGF, CCL13, and VEGFA. Selecting the top five hits of the functional enrichment analysis for biological processes according to the FDR value, these proteins are notably involved in immune cell attraction to tissues, cell signalling and tissue remodelling (Top 5 functions: Lymphocyte chemotaxis, Cytokine-mediated signalling pathway, Killing of cells of another organism, Chemokine-mediated signalling pathway, Cell chemotaxis).

See Fig. 2 for an overview of the heatmap and dendrogram of proteins.

### 3.2 Baseline associations: are HOMA-IR, insulin and HbA1c associated with inflammation?

Significant association of the general cumulative inflammation score was seen with HOMA-IR (F = 7.71, df = 1, p = 0.006), and insulin (F = 5.34, df = 1, p = 0.023) but not HbA1c (F = 1.03, df = 1, p = 0.312). Sensitivity analyses using untransformed data showed essentially similar results (e.g., for HOMA-IR, F = 7.87, df = 1, p = 0.006). The MANCOVA with inflammation sub-scores also revealed significant associations with HOMA-IR (Pillai's trace = 0.16, F(2,102) = 10.02, p < 0.001). Post-hoc analyses revealed that this effect was driven by the sub-score for the cytokine signalling signature (F = 10.77, p < 0.001, p.adj = 0.001), while HOMA-IR was not associated with the immune cell migration protein signature (p = 0.694, p.adj = 0.694). Closer inspection of the proteins involved in the cytokine signalling signature showed that the positive associations were particularly pronounced for IL-10, CCL4, CCL7, CCL3, CCL2, CCL19, IL-6, CXCL10, CCL11, IL-27 and IFN-y (all p.adj<0.05). See Fig. 3 for results of the MANCOVAs and Supplementary Table 1 and Supplementary Fig. 1 for an overview of proteins.

**Fig. 3:**
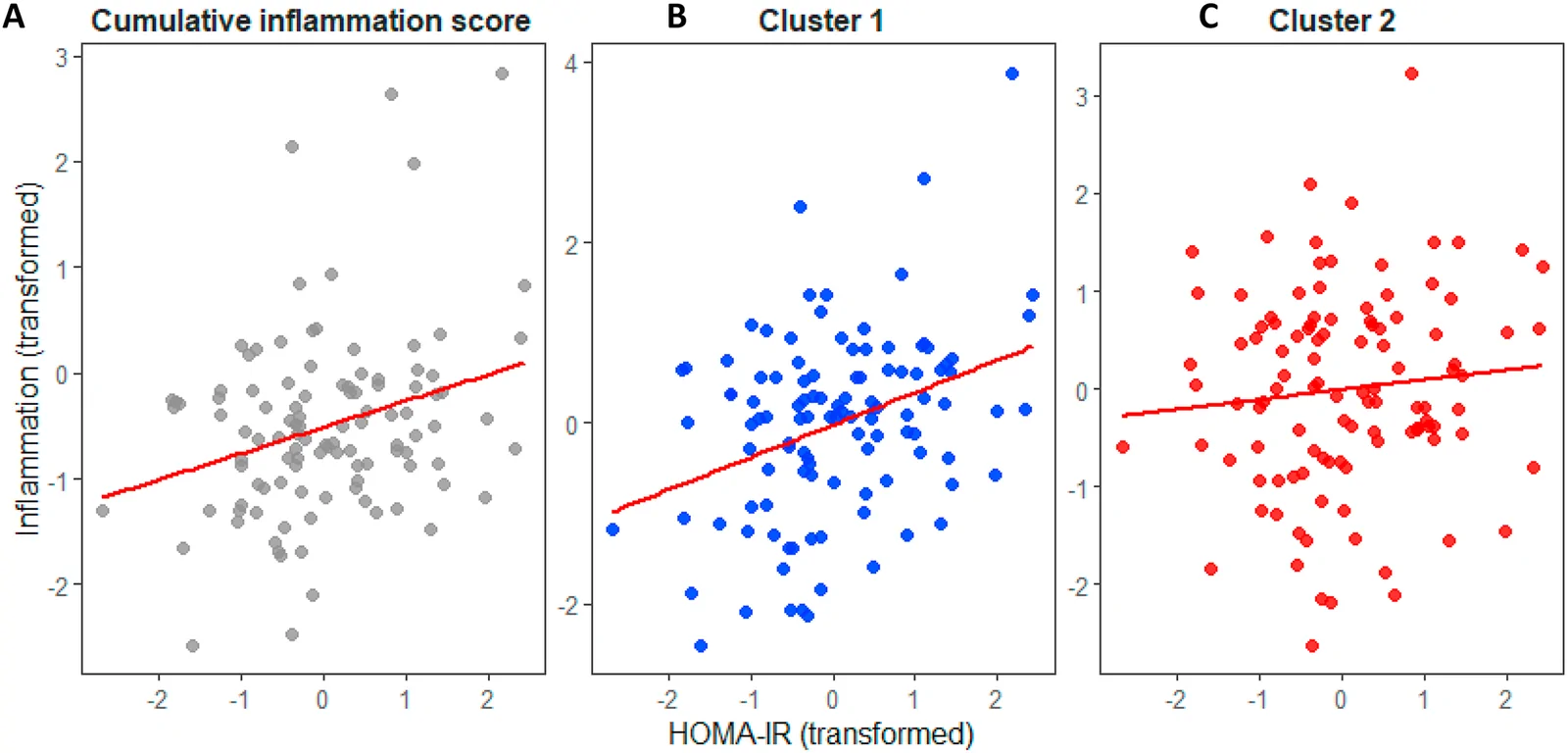
**Association between baseline inflammation scores and HOMA-IR**. Displayed data are transformed values. **A.** Cumulative Inflammation score containing all proteins. **B.** Graph for Cluster 1 (“cytokine signalling”) only. **C.** Graph for Cluster 2 (“immune cell migration) only.

As an exploratory analysis, penalised regression was used to predict HOMA-IR from age, sex, BMI, and the proteins in the cytokine signalling cluster. Model tuning favoured a lasso solution (median and mean α = 1). Across 20 repetitions of nested 10-fold cross-validation, the model achieved a mean out-of-sample (R^2^) of 0.382 (SD = 0.016), indicating that approximately 38% of the variance in HOMA-IR was predicted in held-out observations. BMI (selection frequency = 100%; mean penalised coefficient = 0.079), CCL7 (99.5%, coefficient = 0.077), CCL4 (99.5%, coefficient = 0.100), and IL-10 (94.5%, coefficient = 0.110) were selected in nearly all fitted models, whereas IL-6 (74.5%, coefficient = 0.037) and CCL19 (65.5%, coefficient = 0.020) showed intermediate selection stability, see Supplementary Table 2.

### 3.3 Prediction of IR at follow-up: cross lagged panel analysis

We then assessed whether baseline HOMA-IR could predict inflammation at follow-up, and vice versa, using a bidirectional cross-lagged panel analysis for the total inflammation score and each subcluster. Analyses were also conducted for CRP and IL-6 separately, given their relevance as commonly measured clinical biomarkers (see introduction and methods). A consistent pattern emerged in which baseline HOMA-IR predicted subsequent inflammation more strongly than inflammation predicted subsequent HOMA-IR. Specifically, higher HOMA-IR at baseline predicted higher cumulative inflammation (b = 0.29, β = 0.42, p < 0.001), Cluster 2 scores (b = 0.31, β = 0.34, p < 0.001), and IL-6 levels (b = 0.28, β = 0.24, p = 0.017) at follow-up. In contrast, baseline HOMA-IR did not predict Cluster 1 (b = −0.003, β = −0.003, p = 0.974) or CRP (b = 0.14, β = 0.13, p = 0.194) at follow-up. Notably, CRP showed the opposite directional association, with baseline CRP predicting higher HOMA-IR at follow-up (b = 0.17, β = 0.17, p = 0.021). Baseline-adjusted lagged linear regression sensitivity analyses yielded the same pattern of significant and non-significant cross-lagged associations as the CLPMs (see Supplementary Table 3 and Fig. 4 for the path analysis).

**Fig. 4:**
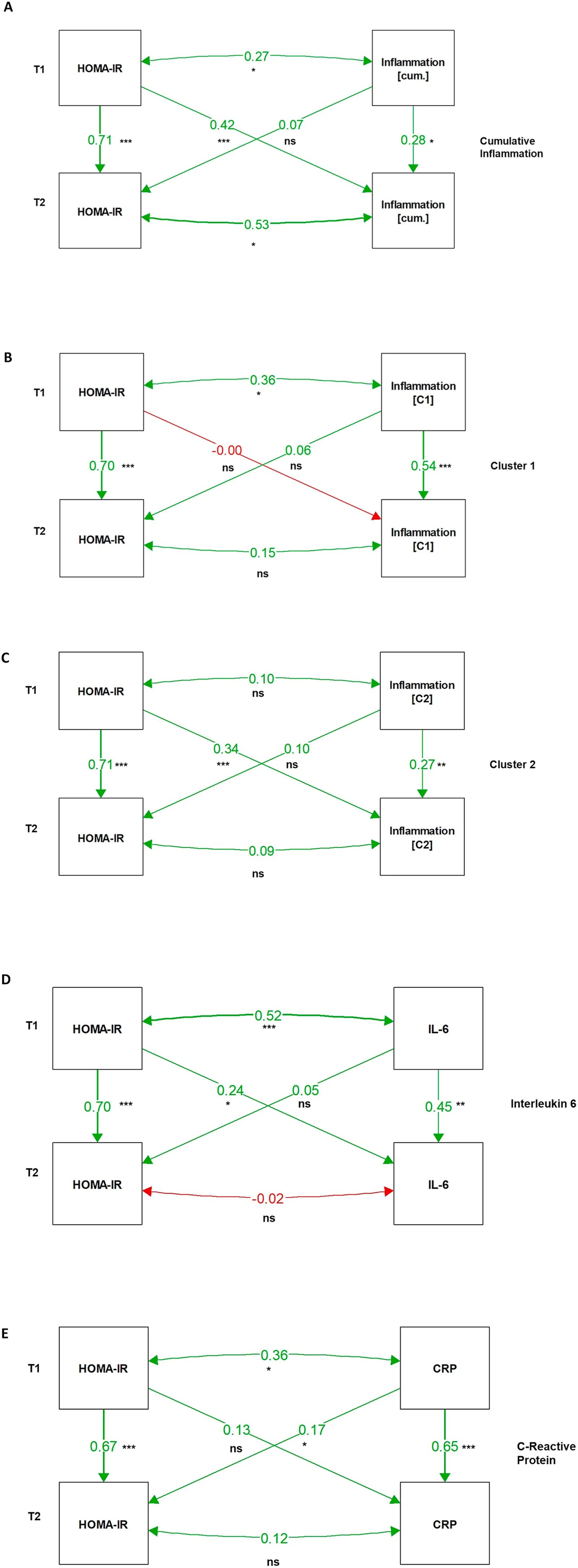
**Cross lagged panel analysis**. The graphs illustrate the dynamic interplay between inflammation and IR over time, both for direct time-lagged effects and the stability of these measures within individuals. Autoregressive (straight) paths show the connection of two variables over time, while cross-lagged (diagonal) paths show the influence of variable A on variable B over time. The numbers displayed above paths are fully standardised path coefficients. Green paths indicate positive relationships, red paths negative relationships. **A.** Total inflammation score. **B.** Cluster 1 (“cytokine signalling” signature); **C.** Cluster 2 (“immune cell migration” signature); **D.** Interleukin (IL)-6; **E.** C Reactive Protein (CRP). (For interpretation of the references to colour in this figure legend, the reader is referred to the Web version of this article.)

### 3.4 Association of inflammation and HOMA-IR with cognitive performance and cognitive decline

Next, we assessed whether inflammation and HOMA-IR were associated with cognitive performance. Baseline correlations revealed significant negative correlations of the cumulative inflammatory index, “cytokine signalling” (Cluster 1) and HOMA-IR with the global cognition (GC) z-score and the working memory (WM) z-score. However, when controlling for age and education, and after FDR adjustment using BH correction, none of the associations remained significant (all p.adj. >0.448). (Fig. 5A, Supplementary Table 4)**.** No significant association emerged for Cluster 1.

**Fig. 5:**
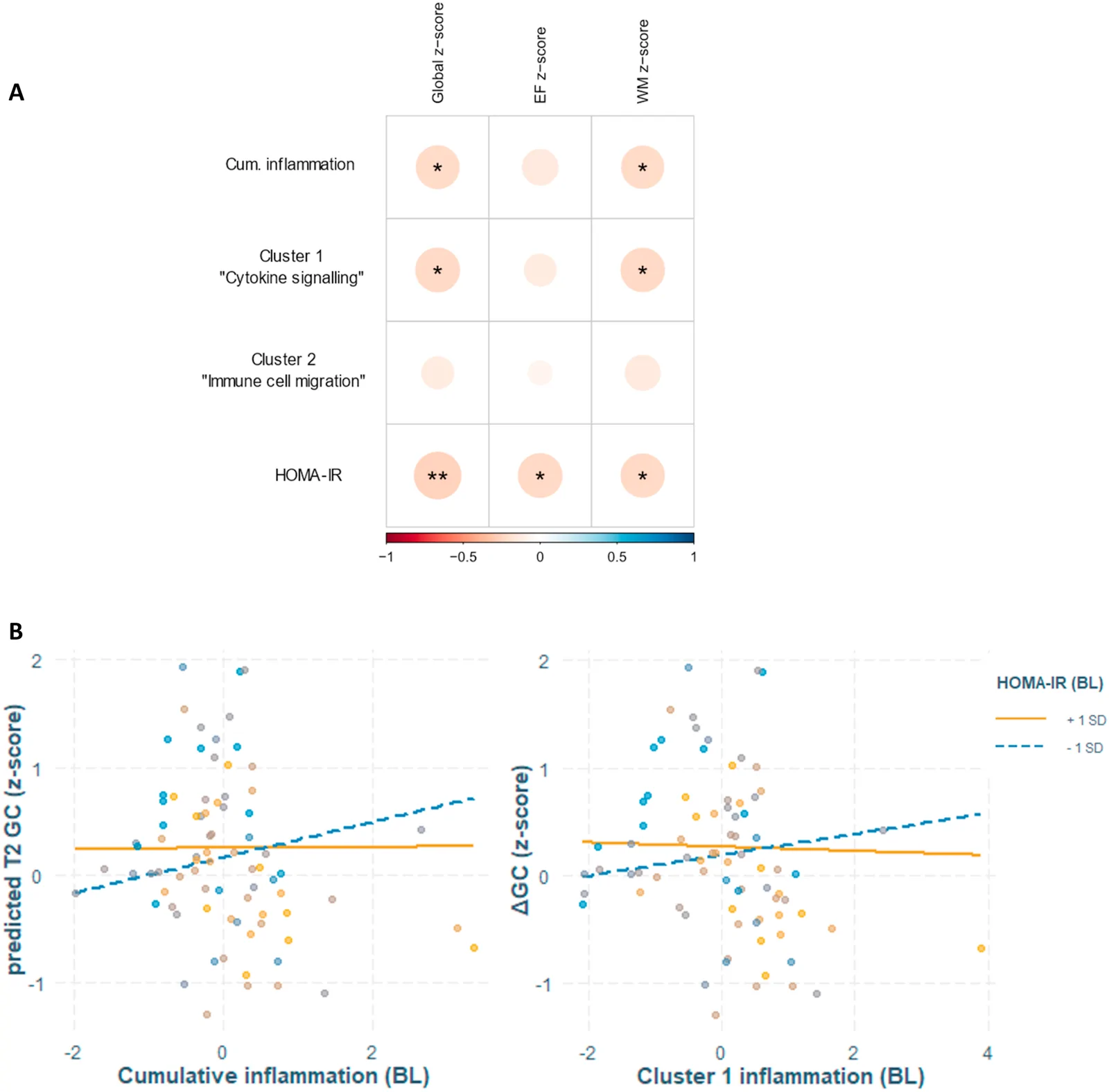
**A.** Correlation of cognitive domains z-scores and inflammation profiles at baseline. **B.** Interaction of cumulative inflammation and HOMA-IR for follow-up GC z-scores, showing effects of high and low HOMA-IR scores (+1SD and -1SD respectively). **C**. Interaction of cumulative inflammation and HOMA-IR for follow-up WM z-scores (trend effect), showing effects of high and low HOMA-IR scores (+1SD and -1SD respectively). Positive scores indicate a better positive performance at T2, negative scores indicate cognitive decline. Robust linear models were used to determine significance, predicted values are adjusted for age, bmi, education and baseline z-scores. Significance: ** = p < 0.01, * = p < 0.05; Blue bar correlation coefficient. (For interpretation of the references to colour in this figure legend, the reader is referred to the Web version of this article.)

### 3.5 Association of baseline inflammation with change in cognitive performance

Cognitive performance at follow-up (including baseline scores for cognition, inflammation, HOMA-IR and age, sex, BMI and education as covariates) was explored next. In robust models, no significant main effects of cumulative inflammation or HOMA-IR emerged for GC, WM, or EF (all p > 0.05), except for cluster 2, where a small main effect emerged for GC (B = 0.08, SE = 0.04, p = 0.021) and for CRP with EF (B = 0.23, SE = 0.08, p = 0.006). The cumulative inflammation × HOMA-IR interaction was significant for GC (B = −0.081; SE = 0.031, p = 0.013) and for Cluster 1 x HOMA-IR for GC (B = −0.058, SE = 0.026, p = 0.028), with the plotted model predictions showing higher T2 performance at lower HOMA-IR (-1SD) relative to higher HOMA-IR (+1SD) (Fig. 5B and C). However, neither association remained significant after correction for multiple testing (p.adj = 0.180 and 0.205 respectively).

No other significant main or interaction effects emerged for Cluster 1, Cluster 2, CRP, or IL-6 across GC, WM, and EF in robust models (all p.adj>0.05).

## 4 Discussion

In this study, we investigated the relationship between inflammation, insulin resistance (IR), and cognitive performance over a one-year follow-up, identifying distinct inflammatory protein signatures associated with IR. At baseline, cytokine-signalling proteins correlated with IR, whereas the immune-cell migration signature did not. However, longitudinally, higher baseline IR was linked to increases in the immune-cell migration signature and IL-6, suggesting that metabolic dysfunction may contribute to progressive immune activation. Among all inflammatory markers, baseline CRP was the only predictor of future IR, underscoring its value as an early indicator of metabolic risk. While baseline associations between cognitive outcomes, HOMA-IR and inflammatory indices existed, none of these measures was significant after adjustment for FDR. Finally, the nominal interactions of HOMA-IR and the cumulative inflammation index in predicting cognitive performance may be indicating value for evaluating both measures when examining their associations with cognitive performance over longer time intervals.

### 4.1 Temporal dynamics of inflammation and IR

Our findings help to disentangle the temporal sequence of different inflammatory signatures. One explanation for the lack of association between immune cell migration-related proteins and IR in our sample at baseline may be that early IR primarily involves cytokine-mediated systemic inflammation rather than localised immune cell infiltration into tissues. Partially in line with our findings, a large population study including 2200 Finnish participants, found that several cytokines were associated with current IR, and that for females (but not males), IL-17 and IL-18 predicted IR over a 4-year follow-up period (Santalahti et al., 2016). Similarly, in a population-based study, a predictive role for IL-6 and a combination of IL-1β and IL-6 was found for the development of T2DM over a follow-up period of approximately 2 years (Spranger et al., 2003). Few studies have assessed chemokines as predictors of IR development, but various cross-sectional studies have reported elevated levels of chemokines in individuals with prediabetes or T2DM, including CCL2, CCL4, CXCL10 and CXCL11 (Pan et al., 2021), which we also observed in our study. It should be acknowledged that the relationship between inflammation and metabolic changes is bidirectional and our data does not permit to reach conclusions on whether tissue inflammation is already present in our sample.

### 4.2 Mechanisms linking IR and tissue inflammation

From a mechanistic perspective, adipose tissue is composed of various cells such as adipocytes, endothelial cells, mesenchymal stromal cells, and immune cells. Immune cells migrate to the tissues and are crucial for normal functioning of our immunological defence mechanisms (Reilly and Saltiel, 2017). During homeostatic conditions (i.e., in lean tissue), resident immune cells clear debris and dying adipocytes and produce cytokines such as IL-5, IL-13, and IL-33, which promote T regulatory cell survival and IL-10 production, thus maintaining an anti-inflammatory environment (Man et al., 2022). However, prolonged inflammatory cytokine signalling may contribute to a microenvironment promoting localised tissue inflammation and initiate further immune cell migration and recruitment of immune cells to the tissues. This process is enhanced when adipocytes become hypertrophic due to excessive fat intake: the adipose tissue produces chemokines to recruit immune cells to the adipose tissue and thus enhances inflammatory processes (Man et al., 2022; Rakotoarivelo et al., 2020).

Once in the adipose tissue, if not resolved, the recruited immune cells (in particular macrophages), can further exacerbate inflammation through the production of inflammatory cytokines (Man et al., 2022). This sustained inflammatory environment may impair glucose metabolism (Li et al., 2022), contributing to IR. In our cross-validated penalised regression, the chemokines CCL4 and CCL7 alongside IL-10 were consistently selected in explaining HOMA-IR, with less convincing selection of cytokines such as IL-6.

The consistent selection of CCL4 and CCL7 in our model aligns with evidence on chemokine-mediated immune-cell recruitment in adipose tissue (Chang and Chen, 2016). As such, in human adipose tissue of obese people, CCL4 expression was increased in insulin-resistant compared with insulin-sensitive individuals independent of BMI (Hardy et al., 2011). Of note, in a small mouse study using high fat diet to induce diabetes, it was shown that inhibition of CCL4 supressed reduced systemic inflammation, improved insulin resistance and delayed the progression of hyperglycaemia in (Chang and Chen, 2021), making CCL4 an interesting target for further research.

In addition, CCL7, a ligand for CCR1, CCR2 and CCR3, has likewise been reported to be increased in visceral adipose tissue in obesity (Huber et al., 2008). By recruiting monocytes/macrophages, CCL7 can enhance inflammatory processes in the vasculature. In their study, and congruent with our results, Chang et al. (2026) found higher plasma levels of CCL7 in a small sample of 10 patients with T2DM compared to controls (Chang et al., 2026). Interestingly, using knock-out models for CCL7, the authors found especially positive effects on vasculopathy in mouse models for diabetes, making it an interesting therapeutic target for diabetic complications (Chang et al., 2026). Seeing this upregulation in our -except for the insulin resistance- healthy subjects, raises the question if CCL7 changes could already be used to mark prediabetic stages.

IL-10, which is usually considered a cytokine with anti-inflammatory properties, was also positively associated with HOMA-IR: this could indicate that its expression and secretion increase with HOMA-IR, suggesting that elevated IL-10 may partly reflect a compensatory response to ongoing adipose inflammation rather than protection from insulin resistance. Interestingly, animal models suggest that T2DM is associated with a hypo-responsiveness to IL-10, thereby enhancing chronic inflammatory conditions (Barry et al., 2016). In mice models for type I diabetes, IL-10 signalling was found to be consistently higher, and was associated with a pathogenic effect including capillary obstruction and cognitive impairment (Sharma et al., 2024), making IL-10 another interesting cytokine to explore further in relation to both cognition and insulin resistance.

### 4.3 CRP as predictive biomarker

In addition to the above-mentioned immune signatures, we also replicated the well-known predictive association between high-sensitive CRP and future IR in our relatively small healthy sample. Various studies have reported cross-sectional associations between CRP and HOMA-IR (e.g. (Shahid et al., 2023; Meng et al., 2007; NAKANISHI et al., 2005)). This association appears robust and was observed in both, larger and smaller cohorts with follow-up periods ranging from 4 to 6.8 years (Park et al., 2009; Yan et al., 2019). We here show that the usefulness of CRP as predictor of IR can even be observed in a relatively short follow-up period such as one year. Supporting this, a recent study reported that CRP measured during pregnancy and at 6-8 weeks postpartum predicted HOMA-IR one year later, highlighting the relevance of this association for various groups including healthy participants (Quansah et al., 2023), making it an interesting biomarker for identifying individuals at higher metabolic risk. This finding also underscores the importance of incorporating CRP measurements in routine clinical assessments, especially in people exhibiting early signs of metabolic syndrome or prediabetes. Stratified treatment strategies based on CRP levels could enable more personalised interventions, such as targeted lifestyle modifications or pharmacological treatments for those with elevated CRP levels.

### 4.4 Inflammation, IR, and cognitive performance

Both inflammation and HOMA-IR were associated with cognitive performance at baseline, but there was no combined (interaction) effect of the two and correction for FDR abolished the significance of the association. In addition, while nominal associations between cognitive performance over one year existed for global cognition, this association was also no longer significant after correction for FDR. Our results contrast with previous studies which have found that classic, pro-inflammatory markers such as IL-6 and insulin resistance predicted cognitive decline rather than improvement over a 10-year follow-up period (Kipinoinen et al., 2022b), particularly for executive functioning and processing speed (Sluiman et al., 2022). While we did not find significant associations within the present follow-up, it is possible that our sample was too small and our follow-up time too short to detect meaningful effects. It is possible that over a longer time span effects for cognitive decline (Gómez-Martínez et al., 2021) could be detected.

### 4.5 Strength and limitations

Our study had several strengths. First, we had a well-characterized sample of healthy people, with a thorough assessment of all three, IR, inflammation (as measured with a proteomic panel) and cognitive performance data, which is a rare combination and enabled us to assess the relationship between those variables. Second, albeit relatively short, the longitudinal follow-up provided us with valuable data on the temporal relationships between those variables: using cross-lagged panel analysis it was possible to assess these effects while controlling for covariance. Third, we used OLINK proteomic data which, in contrast to other studies relying on more readily available inflammation markers such as IL-6 and CRP, provided us with deeper insight into the processes at hand, including cytokine signalling on a broader level and proteins linked to immune cell migration. Of course, to determine effective migration to tissues, functional assays need to be conducted, but our study provides important novel insights to the protein signature alterations relevant to IR. Our study has several limitations. First, it is possible that our follow-up period was too short to show any predictive effects and/or our cohort was too small to detect effects and while we retained 74% of the participants, the attrition rate may have affected the longitudinal outcomes in this study. Second, while our cluster analyses revealed useful patterns, it should be noted that in the “cytokine signalling signature”, various chemokines were also included. In a larger data set, an even more fine-grained approach could be useful. Other variables not considered in the models could have influenced the results (i.e., dietary patterns, physical activity, depression levels). Finally, these results have to be considered with caution and replicated in other samples of participants.

## 5 Conclusions

In summary, in our sample, the disentanglement of inflammatory processes suggests a progressive nature of inflammatory responses preceding IR, where cytokine signalling may act as a precursor to more advanced inflammatory processes. Understanding this sequence in more depth may aid in identifying critical windows for lifestyle and/or anti-inflammatory interventions, to disrupt this cascade and diminish tissue migration of immune cells, thereby improving long-term outcomes. The chemokines CCL4 and CCL7, and IL-10 as well as CRP should be explored further for their role as potential targets for diabetes prevention. Although baseline HOMA-IR and inflammation were associated with cognitive performance, the effects were small and did not survive correction for FDR, highlighting the need for future studies to assess longer follow-up periods. Furthermore, future investigations should extend inflammatory signatures beyond the classical proteins (i.e., IL-6, CRP) to gain a better understanding of pathways that could be beneficial for delaying or treating IR.

## CRediT authorship contribution statement

**Carmen Schiweck:** Conceptualization, Formal analysis, Visualization, Writing – original draft, Writing – review & editing. **Judith Gruber:** Investigation, Data curation, Writing – original draft, Writing – review & editing. **Janita Bralten:** Writing – review & editing. **Giuseppe Fanelli:** Writing – review & editing. **Indira Paz-Graniel:** Writing – review & editing. **Veerle van Gils:** Writing – review & editing. **Carlos Gomez-Martinez:** Writing – review & editing. **Jordi Salas-Salvadó:** Writing – review & editing. **Jeffrey Glennon:** Writing – review & editing. **Geert Poelmans:** Writing – review & editing. **Barbara Franke:** Funding acquisition, Writing – review & editing. **Andreas Reif:** Funding acquisition, Writing – review & editing. **Silke Matura:** Conceptualization, Writing – original draft, Writing – review & editing.

## Declaration of generative AI and AI-assisted technologies in the manuscript preparation process

During the preparation of this work the authors used ChatGPT in order to improve wording, coding and readability. After using this tool/service, the authors reviewed and edited the content as needed and take full responsibility for the content of the published article.

## Funding

This project has received funding from the European Union’s Horizon 2020 research and innovation programme under grant agreement No 847879. Jordi Salas-Salvadó, is partially supported by ICREA under the ICREA Academia program. Indira Paz-Graniel was granted by the Sara Borrell postdoctoral fellowship (CD24/00015 – Instituto de Salud Carlos III). Andreas Reif was also funded by the Hessian Ministry of Science and Arts (HMWK) and LOEWE program (LOEWE center DYNAMIC). We thank Nicole Ziegler (Fraunhofer Institute) and her team for the excellent collaboration and execution of the OLINK analyses.

## Declaration of competing interest

The work reported in this article received funding from the European Commission, as detailed in the Funding section. Barbara Franke has received lecture fees and travel reimbursement from MEDICE. Andreas Reif has received consulting or advisory fees and travel reimbursement from Janssen Pharmaceuticals, Takeda, Sage Therapeutics, and Cyclerion Therapeutics; and consulting or advisory fees, lecture fees, and travel reimbursement from Boehringer Ingelheim and Medice. The remaining authors declare that they have no other known competing financial interests or personal relationships that could have appeared to influence the work reported in this article.
