# Supplementary material for: Inflammatory protein signatures as predictors of insulin resistance and cognitive function: a one-year longitudinal study in the mPRIME cohort
Source: Brain Behav Immun Health. 2026 Sep 22;57:101366. doi: 10.1016/j.bbih.2026.101366 (PMC13634109; doi:10.1016/j.bbih.2026.101366)
Supplement: Supplementary file 1 [file mmc1.docx]

**Supplementary information**

**Supplementary Table 1.** Post-hoc results on association of HOMA-IR with proteins of the cytokine signalling cluster (Cluster 1), adjusted for age, sex, and BMI.

| **Protein** | **F** | **p** | **p.adj** |
| --- | --- | --- | --- |
| IL-10 | 12.316 | 0.001 | 0.010 |
| CCL4 | 12.001 | 0.001 | 0.010 |
| CCL7 | 10.621 | 0.002 | 0.013 |
| CCL3 | 9.737 | 0.002 | 0.015 |
| CCL2 | 8.255 | 0.005 | 0.025 |
| CCL19 | 7.493 | 0.007 | 0.030 |
| CXCL10 | 7.096 | 0.009 | 0.032 |
| IL-6 | 6.780 | 0.011 | 0.033 |
| IFNG | 6.455 | 0.013 | 0.035 |
| IL-27 | 5.837 | 0.017 | 0.044 |
| CCL11 | 5.514 | 0.021 | 0.047 |
| CCL8 | 4.126 | 0.045 | 0.093 |
| FLT3LG | 3.050 | 0.084 | 0.161 |
| TGFA | 2.816 | 0.096 | 0.172 |
| TNF | 2.244 | 0.137 | 0.218 |
| CSF3 | 2.220 | 0.139 | 0.218 |
| CSF1 | 1.700 | 0.195 | 0.287 |
| LTA | 1.449 | 0.231 | 0.321 |
| IL-15 | 1.315 | 0.254 | 0.334 |
| TNFSF10 | 1.176 | 0.281 | 0.337 |
| IL-18 | 1.164 | 0.283 | 0.337 |
| CXCL8 | 0.836 | 0.363 | 0.405 |
| MMP12 | 0.803 | 0.372 | 0.405 |
| IL-17C | 0.072 | 0.788 | 0.804 |
| CXCL9 | 0.062 | 0.804 | 0.804 |

**Supplementary Figure 1**. Baseline association of individual proteins with HOMA-IR. Data is transformed as described in the methods section and regression lines are **not** adjusted for age, sex and BMI.

**Supplementary Table 2. Predictor stability in repeated nested cross-validated penalised regression predicting baseline HOMA-IR.**

| **Predictor** | **Selection frequency (%)** | **Mean penalised coefficient** | **Mean coefficient when selected** |
| --- | --- | --- | --- |
| **BMI** | **100** | **0.079** | **0.079** |
| **CCL4** | **99.5** | **0.1** | **0.101** |
| **CCL7** | **99.5** | **0.077** | **0.078** |
| **IL-10** | **94.5** | **0.111** | **0.117** |
| IL-6 | 74.5 | 0.038 | 0.05 |
| CCL19 | 65.5 | 0.021 | 0.031 |
| FLT3LG | 44 | 0.012 | 0.028 |
| CCL3 | 42 | 0.012 | 0.028 |
| CXCL9 | 40 | -0.036 | -0.09 |
| CCL11 | 34.5 | 0.01 | 0.028 |
| CCL2 | 22.5 | 0.005 | 0.023 |
| IFNG | 9.5 | 0.003 | 0.028 |
| CXCL10 | 7 | 0.003 | 0.046 |
| CXCL8 | 5.5 | -0.004 | -0.075 |
| CCL8 | 3 | 0.001 | 0.038 |
| CSF3 | 3 | 0 | 0.015 |
| IL-15 | 2.5 | -0.001 | -0.029 |
| Sex | 2 | 0 | 0.006 |
| IL-17C | 2 | 0 | -0.012 |
| MMP12 | 1 | 0 | -0.016 |
| Age | 0.5 | 0 | 0 |
| TNFSF10 | 0.5 | 0 | 0.023 |
| LTA | 0.5 | 0 | 0.045 |
| IL-27 | 0 | 0 | ns |
| TGFA | 0 | 0 | ns |
| IL-18 | 0 | 0 | ns |
| TNF | 0 | 0 | ns |
| CSF1 | 0 | 0 | ns |

***Note.*** *Selection frequency indicates the percentage of outer cross-validation models in which the predictor had a coefficient other than 0. Mean penalised coefficients were calculated across all models, including coefficients shrunk to 0. “ns” indicates that the predictor was never selected. Penalised coefficients are descriptive and do not represent effect estimates. Predictors with selection frequency ≥80% are shown in bold.*

**Supplementary Table 3. Cross-lagged panel models and baseline-adjusted lagged linear regressions**

| **T2 Variable** | **T1 Variable** | **CLPM b** | **CLPM β** | **CLPM p** | **Lagged regression b** | **Lagged regression p** |
| --- | --- | --- | --- | --- | --- | --- |
| **Cumulative inflammation T2** | **HOMA-IR T1** | **0.287** | **0.418** | **<0.001** | **0.287** | **<0.001** |
| HOMA-IR T2 | Cumulative inflammation T1 | 0.079 | 0.070 | 0.382 | 0.079 | 0.400 |
| Cluster 1 T2 | HOMA-IR T1 | −0.003 | −0.003 | 0.974 | −0.003 | 0.978 |
| HOMA-IR T2 | Cluster 1 T1 | 0.059 | 0.058 | 0.506 | 0.059 | 0.498 |
| **Cluster 2 T2** | **HOMA-IR T1** | **0.313** | **0.343** | **<0.001** | **0.313** | **0.001** |
| HOMA-IR T2 | Cluster 2 T1 | 0.094 | 0.100 | 0.150 | 0.094 | 0.209 |
| **IL-6 T2** | **HOMA-IR T1** | **0.280** | **0.240** | **0.017** | **0.280** | **0.029** |
| HOMA-IR T2 | IL-6 T1 | 0.062 | 0.051 | 0.527 | 0.062 | 0.586 |
| CRP T2 | HOMA-IR T1 | 0.138 | 0.133 | 0.194 | 0.138 | 0.131 |
| **HOMA-IR T2** | **CRP T1** | **0.171** | **0.167** | **0.021** | **0.171** | **0.048** |

***Note****. b = unstandardized coefficient; β = standardized coefficient. Lagged regression models adjusted each T2 outcome for its corresponding T1 value. CLPMs additionally modelled the covariance between the T1 variables and the residual covariance between the T2 outcomes. The CLPMs and lagged regressions yielded identical point estimates but slightly different p-values because the CLPMs accounts for the joint covariance structure and used robust standard errors. Significant results are printed in bold.*

**Supplementary Table 4**. Correlation coefficents for Cognitive performance with inflammation and HOMA-IR at baseline. R (Pearson) represents the unadjusted correlation coefficients; Partial r represents the values adjusted for age and education as indicated. P.adj is corrected with Benjamini Hochberg Correction.

| **Outcome / Predictor** | **r (Pearson)** | **t** | **p** | **Partial r (age + education)** | **p** | **p.adj** |
| --- | --- | --- | --- | --- | --- | --- |
|  |  |  |  |  |  |  |
| **Global Cognition** |  |  |  |  |  |  |
| Cumulative score | **−0.196** | **−2.056** | **0.042** | −0.065 | 0.510 | 0.701 |
| Cluster 1 | **−0.207** | **−2.184** | **0.031** | −0.052 | 0.600 | 0.701 |
| Cluster 2 | −0.112 | −1.165 | 0.247 | −0.075 | 0.447 | 0.701 |
| HOMA-IR | **−0.256** | **−2.716** | **0.008** | −0.176 | 0.075 | 0.448 |
| **Working Memory** |  |  |  |  |  |  |
| Cumulative score | **−0.200** | **−2.100** | **0.038** | −0.062 | 0.529 | 0.701 |
| Cluster 1 | **−0.213** | **−2.247** | **0.027** | −0.057 | 0.569 | 0.701 |
| Cluster 2 | −0.139 | −1.445 | 0.152 | −0.095 | 0.339 | 0.701 |
| HOMA-IR | **−0.221** | **−2.325** | **0.022** | −0.123 | 0.216 | 0.701 |
| **Executive Function** |  |  |  |  |  |  |
| Cumulative score | −0.129 | −1.340 | 0.183 | −0.056 | 0.571 | 0.701 |
| Cluster 1 | −0.106 | −1.095 | 0.276 | −0.010 | 0.917 | 0.917 |
| Cluster 2 | −0.062 | −0.637 | 0.525 | −0.046 | 0.643 | 0.701 |
| **HOMA-IR** | **−0.227** | **−2.386** | **0.019** | −0.186 | 0.059 | 0.448 |
